# Clinical and Genetic Characteristics of 18 Patients from 13 Japanese Families with CRX-associated retinal disorder: Identification of Genotype-phenotype Association

**DOI:** 10.1038/s41598-020-65737-z

**Published:** 2020-06-12

**Authors:** Yu Fujinami-Yokokawa, Kaoru Fujinami, Kazuki Kuniyoshi, Takaaki Hayashi, Shinji Ueno, Atsushi Mizota, Kei Shinoda, Gavin Arno, Nikolas Pontikos, Lizhu Yang, Xiao Liu, Hiroyuki Sakuramoto, Satoshi Katagiri, Kei Mizobuchi, Taro Kominami, Hiroko Terasaki, Natsuko Nakamura, Shuhei Kameya, Kazutoshi Yoshitake, Yozo Miyake, Toshihide Kurihara, Kazuo Tsubota, Hiroaki Miyata, Takeshi Iwata, Kazushige Tsunoda, Japan Eye Genetics Consortium, Toshihide Nishimura, Yoshihide Hayashizaki, Mineo Kondo, Nobuhiro Shimozawa, Masayuki Horiguchi, Shuichi Yamamoto, Manami Kuze, Nobuhisa Naoi, Shigeki Machida, Yoshiaki Shimada, Makoto Nakamura, Takashi Fujikado, Yoshihiro Hotta, Masayo Takahashi, Kiyofumi Mochizuki, Akira Murakami, Hiroyuki Kondo, Susumu Ishida, Mitsuru Nakazawa, Tetsuhisa Hatase, Tatsuo Matsunaga, Akiko Maeda, Kosuke Noda, Atsuhiro Tanikawa, Syuji Yamamoto, Hiroyuki Yamamoto, Makoto Araie, Makoto Aihara, Toru Nakazawa, Tetuju Sekiryu, Kenji Kashiwagi, Kenjiro Kosaki, Carninci Piero, Takeo Fukuchi, Atsushi Hayashi, Katsuhiro Hosono, Keisuke Mori, Kouji Tanaka, Koichi Furuya, Keiichirou Suzuki, Ryo Kohata, Yasuo Yanagi, Yuriko Minegishi, Daisuke Iejima, Akiko Suga, Brian P. Rossmiller, Yang Pan, Tomoko Oshima, Mao Nakayama, Yu Teruyama, Megumi Yamamoto, Naoko Minematsu, Hideko Sanbe, Daisuke Mori, Yusuke Kijima, Go Mawatari, Kentaro Kurata, Norihiro Yamada, Masayosi Itoh, Hideya Kawaji, Yasuhiro Murakawa

**Affiliations:** 1 Laboratory of Visual Physiology, Division of Vision Research, National Institute of Sensory Organs, National Hospital Organization Tokyo Medical Center, Tokyo, 152-8902 Japan; 2 Department of Health Policy and Management, Keio University School of Medicine, Tokyo, Japan; 3 Division of Public Health, Yokokawa Clinic, Suita, 564-0083 Japan; 4 Department of Ophthalmology, Keio University School of Medicine, Tokyo, 160-8582 Japan; 5 UCL Institute of Ophthalmology, London, EC1V 9EL UK; 6 Moorfields Eye Hospital, London, EC1V 2PD UK; 7 Department of Ophthalmology, Kindai University Faculty of Medicine, Osaka-Sayama, 589-8511 Japan; 8 Department of Ophthalmology, The Jikei University School of Medicine, Tokyo, 105-8461 Japan; 9 Department of Ophthalmology, Nagoya University Graduate School of Medicine, Nagoya, 466-8550 Japan; 10 Department of Ophthalmology, Teikyo University, Tokyo, 173-8605 Japan; 11 Department of Ophthalmology, Saitama Medical University, Saitama, 350-0495 Japan; 12 North East Thames Regional Genetics Service, UCL Great Ormond Street Institute of Child Health, Great Ormond Street NHS Foundation Trust, London WC1N 1EH, London, UK; 13 Southwest Hospital/Southwest Eye Hospital, Third Military Medical University, Chongqing, 400030 China; 14 Department of Ophthalmology, The University of Tokyo, Tokyo, 113-8654 Japan; 15 Department of Ophthalmology, Nippon Medical School Chiba Hokusoh Hospital, Inzai, 270-1694 Japan; 16 Division of Molecular and Cellular Biology, National Institute of Sensory Organs, National Hospital Organization Tokyo Medical Center, Tokyo, 152-8902 Japan; 17 Aichi Medical University, Nagakute, 480-1195 Japan; 18 Kobe Eye Center, Next Vision, Kobe, 650-0047 Japan; 19 Department of Healthcare Quality Assessment, University of Tokyo, Tokyo, 113-8655 Japan; 20 Department of Translational Medicine Informatics, St. Marianna University School of Medicine, Kawasaki, 216-8511 Japan; 21 RIKEN Preventive Medicine and Diagnosis Innovation Program, Wako, 351-0198 Japan; 22 Department of Ophthalmology, Mie University Graduate School of Medicine, Mie, 514-8507 Japan; 23 National Institutes of Biomedical Innovation, Health and Nutrition, Tsukuba, 305-0843 Japan; 24 Department of Ophthalmology, Fujita Health University School of Medicine, Toyoake, 470-1192 Japan; 25 Department of Ophthalmology and Visual Science, Chiba University Graduate School of Medicine, Chiba, 263-8522 Japan; 26 Department of Ophthalmology, Matsusaka Central General Hospital, Matsusaka, 515-8566 Japan; 27 Department of Ophthalmology, University of Miyazaki, Miyazaki, 889-2192 Japan; 28 Saitama Medical Center, Dokkyo Medical University, Koshigaya, Saitama, 345-8555 Japan; 29 Fujita Health Univ Banbuntane Hosp, Nagoya, 454-8509 Japan; 30 Department of Ophthalmology, Kobe University Hospital, Kobe, 650-0017 Japan; 31 Osaka Univ Medical School, Suita, 565-0871 Japan; 32 Hamamatsu University School of Medicine, Hamamatsu, Japan; 33 Riken Center for Developmental Biology, Kobe, 650-0047 Japan; 34 Department of Ophthalmology Gifu University Graduate School of Medicine, Gifu, 501-1112 Japan; 35 Department of Ophthalmology, Juntendo University Faculty of Medicine, Tokyo, 113-8431 Japan; 36 Department of Ophthalmology, University of Occupational and Environmental Health, Kitakyuusyuu, 807-8556 Japan; 37 Laboratory of Ocular Cell Biology and Visual Science, Department of Ophthalmology, Faculty of Medicine and Graduate School of Medicine, Hokkaido University, Sapporo, Hokkaido 060-0808 Japan; 38 Hirosaki University Graduate School of Medicine, Hirosaki, 036-8562 Japan; 39 Graduate School of Medical and Dental Sciences, Niigata University, Niigata, 951-8510 Japan; 40 Division of Hearing and Balance Research, National Institute of Sensory Organs, National Hospital Organization, Tokyo Medical Center, Tokyo, 158-8531 Japan; 41 Hitoshi Ophthalmology Clinic, Nishinomiya, 663-8184 Japan; 42 Kanto Central Hospital of the Mutual Aid Association of Public School Teachers, Tokyo, 158-8531 Japan; 43 Department of Ophthalmology, The University of Tokyo, Tokyo, 113-8654 Japan; 44 Department of ophthalmology, Graduate School of Medicine, Tohoku University, Sendai, 980-8577 Japan; 45 Department of Ophthalmology, Fukushima Medical University School of Medicine, Fukushima, 960-1247 Japan; 46 Department of Ophthalmology, University of Yamanashi, Chuo, 409-3898 Japan; 47 Center for Medical Genetics, Keio University School of Medicine, Tokyo, 160-8582 Japan; 48 Division of Genomic Technologies, Laboratory for Transcriptome Technology, RIKEN Center for Integrative Medical Sciences, Yokohama, 230-0045 Japan; 49 Division of Ophthalmology and Visual Science, Graduate School of Medical and Dental Sciences, Niigata University, Niigata, 950-2181 Japan; 50 Department of Radiological Sciences, Graduate School of Medicine and Pharmaceutical Sciences, University of Toyama, Toyama, 930-0194 Japan; 51 Department of Ophthalmology, International University of Health and Welfare, Nasu-shiobara, 329-2763 Japan; 52 Department of Ophthalmology, Nihon University Hospital, Tokyo, 101-8309 Japan; 53 Institute for Advanced Co-Creation Studies, Osaka University, Suita, 565-0871 Japan; 54 Department of Ophthalmology, Asahikawa Medical University, Asahikawa, 078-8802 Japan; 55 Graduate School of Agricultural and Life Sciences, The University of Tokyo, Tokyo, 113-8654 Japan; 56 RIKEN Preventive Medicine and Diagnosis Innovation Program, Yokohama, 230-0045 Japan

**Keywords:** Disease genetics, Retinal diseases

## Abstract

Inherited retinal disorder (IRD) is a leading cause of blindness, and *CRX* is one of a number of genes reported to harbour autosomal dominant (AD) and recessive (AR) causative variants. Eighteen patients from 13 families with CRX-associated retinal disorder (*CRX*-RD) were identified from 730 Japanese families with IRD. Ophthalmological examinations and phenotype subgroup classification were performed. The median age of onset/latest examination was 45.0/62.5 years (range, 15–77/25–94). The median visual acuity in the right/left eye was 0.52/0.40 (range, −0.08–2.00/−0.18–1.70) logarithm of the minimum angle of resolution (LogMAR) units. There was one family with macular dystrophy, nine with cone-rod dystrophy (CORD), and three with retinitis pigmentosa. *In silico* analysis of *CRX* variants was conducted for genotype subgroup classification based on inheritance and the presence of truncating variants. Eight pathogenic *CRX* variants were identified, including three novel heterozygous variants (p.R43H, p.P145Lfs*42, and p.P197Afs*22). A trend of a genotype-phenotype association was revealed between the phenotype and genotype subgroups. A considerably high proportion of *CRX*-RD in ADCORD was determined in the Japanese cohort (39.1%), often showing the mild phenotype (CORD) with late-onset disease (sixth decade). Frequently found heterozygous missense variants located within the homeodomain underlie this mild phenotype. This large cohort study delineates the disease spectrum of *CRX*-RD in the Japanese population.

## Introduction

Inherited retinal disorder (IRD) is one of the major causes of blindness in developed countries in both adults and children^1^ and includes retinitis pigmentosa (RP), cone/cone-rod dystrophy (CORD), Stargardt disease (STGD), macular dystrophy (MD), Leber congenital amaurosis (LCA) and others. Different inheritance patterns are found in IRD: autosomal dominant (AD), autosomal recessive (AR), X-linked, and mitochondrial inheritance^2–9^. For instance, different inheritance patterns result in different phenotypes (*GUCY2D, BEST1, PROM1*) in some genes, while other genes are associated with different phenotypes with similar inheritance patterns (*ABCA4, PRPH2, RPGR, KCNV2, GNGA3, CNGB3*)^8–17^.

*CRX*, denoted as a cone–rod homeobox-containing gene (OMIM: 602225) with high homology to the OTX family of homeobox genes, is located on 19q13.33 and contains four exons encoding a 299-amino acid homeodomain transcription factor crucial for the development and survival of photoreceptors^18^. Animal experiments have proven that *CRX* is predominantly expressed in vertebrate photoreceptor cells of the retina and pinealocytes of the pineal gland^19,20^, playing a significant role in the differentiation and maintenance of photoreceptor cells by synergistic interactions with other transcription factors, such as neural retina-specific leucine zipper protein (NRL), retinal homeobox protein (RAX), and nuclear receptor subfamily 2 group E member 3 (NR2E3)^19,21,22^.

A locus and gene for ADCORD (CORD2) was first mapped and identified as *CRX* in 1994^18,23^. Since then, over 90 variants in the *CRX* gene have been associated with a wide range of different phenotypes of IRDs, including CORD, LCA, MD, and RP (The Human Gene Mutation Database; http://www.hgmd.cf.ac.uk/ac/index.php; accessed on 1 August 2018)^24–35^. The predominant mode of inheritance in reported families is AD, and in a few patients, including Japanese with RP or LCA caused by homozygous *CRX* variants was reported^29,36^.

Studies of *CRX*-associated retinal disorder (*CRX*-RD) have often been separately conducted for each phenotype, such as CORD or RP/LCA^16,24,26,27,32,36^; thus, it has been hard to comprehensively understand the disorder in consideration of different phenotypes and different modes of inheritance. To solve such a problem, large cohort studies with standardized clinical and genetic investigations for IRDs are required.

The purpose of this study was to characterize the clinical and molecular genetic features of *CRX*-RD in a nationwide large cohort of Japanese subjects diagnosed with IRD.

## Results

### Participants

**Figure 1:**
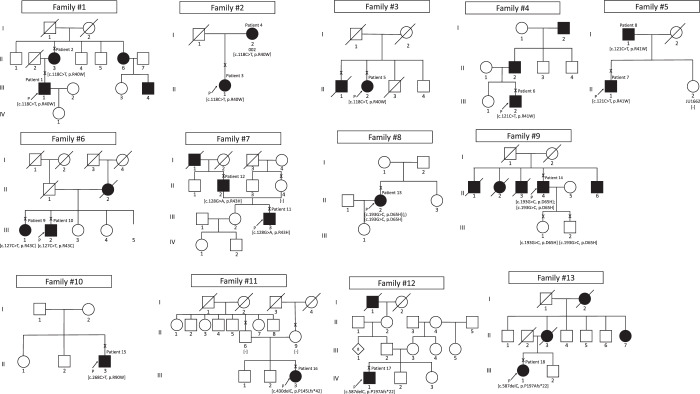
Pedigrees of 13 Japanese families with inherited retinal disorder harbouring *CRX* variants. The solid squares and circles (men and women, respectively) represent the affected subjects, and the white icons represent the unaffected family members. The slash symbol shows deceased individuals. The generation number is noted on the left. The proband is marked by an arrow, and the clinically investigated individuals are indicated by a cross.

Eighteen affected subjects from 13 Japanese families with a clinical diagnosis of IRD and harbouring *CRX* variants were identified in this study. The detailed clinical information is provided in Table 1, and the pedigrees of the 13 families are demonstrated in Fig. 1.

**Table 1:** Demographics and detected variants in 18 Japanese patients from 13 families with CRX-associated retinal disorder (CRX-RD).

Family No.	Patient No.	JEGC consortium ID	Inheritance based on family history	Sex	Age (at latest examination)	Onset	Chief complaint	Refractive errors		BCVA (LogMAR unit)		Phenotype subgroup	Molecularly raised inheritance	CRX variants
								RE (dioptre)	LE (dioptre)	RE	LE			
1	Patient 1 (1-III:1)	TMC-001-001	AD	M	44	35	Reduced visual acuity	−5.5	−5.0	1.1	0.1	CORD	AD	c.118C>T, p.R40W
1	Patient 2 (1-II:3)	TMC-001-002	AD	F	72	NA	Photophobia	−0.5	−0.5	0	0	CORD	AD	c.118C>T, p.R40W
2	Patient 3 (2-II:1)	JU-001-001	AD	F	71	56	Reduced visual acuity	0.5	−1.5	1.0	0.2	CORD	AD	c.118C>T, p.R40W
2	Patient 4 (2-I:2)	JU-001-002	AD	F	94	30	Reduced visual acuity	0.0	0.0	2.0	CF	CORD	AD	c.118C>T, p.R40W
3	Patient 5 (3-II:2)	KDU-001-001	Unknown	F	76	NA	Reduced visual acuity	−3.5	NA	0.4	1.7	CORD	AD	c.118C>T, p.R40W
4	Patient 6 (4-III:2)	NU-001-001	AD	M	32	NA	Photophobia	−0.5	−0.5	0.52	0.52	CORD	AD	c.121C>T, p.R41W
5	Patient 7 (5-II:1)	JU-002-001	AD	M	63	NA	Reduced visual acuity	+1.0	+1.5	0.15	0.4	CORD	AD	c.121C>T, p.R41W
5	Patient 8 (5-I:1)	JU-002-002	AD	M	88	60	Night blindness	0	−0.5	1.15	1.22	CORD	AD	c.121C>T, p.R41W
6	Patient 9 (6-III:1)	KDU-002-001	AD	F	80	75	Reduced visual acuity	0.0	0.0	0.52	0.82	CORD	AD	c.127C>T, p.R43C
6	Patient 10 (6-III:2)	KDU-002-002	AD	M	83	77	Reduced visual acuity	+2.0	+2.0	0.7	0.7	CORD	AD	c.127C>T, p.R43C
7	Patient 11 (7-III:3)	TMC-002-001	AD	M	35	31	Central visual field loss	NA	NA	0.22	0.4	MD	AD	*c.128G>A, p.R43H*
7	Patient 12 (7-II:2)	TMC-002-002	AD	M	63	62	No symptoms	NA	NA	−0.08	−0.08	MD	AD	*c.128G>A, p.R43H*
8	Patient 13 (8-II:2)	TMC-003-001	Sporadic	F	41	37	Reduced visual acuity	+1.5	−0.5	0.7	0.4	RP	AR	c.193G>C, p.D65H/c.193G>C, p.D65H
9	Patient 14 (9-II:4)	KDU-003-001	AR	M	50	NA	NA	−2.0	NA	0.82	LP	RP	AR	c.193G>C, p.D65H/c.193G>C, p.D65H
10	Patient 15 (10-II:3)	JU-003-001	Sporadic	M	55	45	Reduced visual acuity	−3.5	−3.5	0.22	−0.18	CORD	AD	c.268C>T, p.R90W
11	Patient 16 (11-III:3)	KDU-004-001	Sporadic	F	25	15	Night blindness	−3.5	−5.0	0.52	0.7	RP	AD (de novo)	c.430delC, p.P145Lfs*42
12	Patient 17 (12-IV:1)	TU-001-001	Unknown	M	51	45	Reduced visual acuity	−2.5	−2.5	0	−0.08	CORD	AD	c.587delC, p.P197Afs*22
13	Patient 18 (13-III:1)	TMC-004-001	AD	F	62	30	Reduced visual acuity	−1.0	−1.0	0.82	0.82	CORD	AD	c.587delC, p.P197Afs*22

AD = autosomal dominant; AR = autosomal recessive; CORD = cone-rod dystrophy; F = female; CF = counting finger; LCA = Leber congenital amaurosis; LE = left eye; LogMAR BCVA = best-corrected Snellen visual acuity converted to the logarithm of the minimum angle of resolution visual acuity; LP = light perception; M = male; MD = macular dystrophy; No.=number; NA = not available; RE = right eye; RP = retinitis pigmentosa. All affected and unaffected subjects are originally from Japan and any mixture with other ethnicity was not reported. Age was defined as the age when the latest examination was performed. The age of onset was defined as either the age at which visual loss was first noted by the patient or when an abnormal retinal finding was first detected. Phenotype subgroup was defined based on clinical manifestations such as onset of disease, natural course, lesioned part on retinal imaging, and pattern of retinal dysfunction: LCA (including early-onset RP), a severe retinal dystrophy with early onset (<10 years) and extinguished retinal function; RP (including rod-cone dystrophy), a progressive retinal dystrophy often initially presenting peripheral atrophy with generalized rod dysfunction greater than cone dysfunction; CORD, a progressive retinal dystrophy often initially presenting macular atrophy with generalized cone dysfunction greater than rod dysfunction; MD, a progressive retinal dystrophy presenting macular atrophy with confined macular dysfunction despite no abnormalities in generalized cone and rod function. Syndromic findings of central nervous system abnormalities (described as multiple sclerosis-like changes) were reported in Patient 8.

There were seven families with clear AD family history (7/13, 53.8%; Families 1, 2, 4, 5, 6, 7, 13), one family with a family history consistent with AR inheritance with affected siblings born to unaffected parents (1/13, 7.7%; Family 9), and 3 sporadic cases (3/13, 23.1%; Families 8, 10, 11). Two families lacked clear family data: one with unknown parental affected status (Family 3) and the other with the presence of an affected deceased paternal great grandfather (Family 12). Consanguineous marriage was not clearly reported in all families.

There were eight affected females (8/18, 44.4%) and ten affected males (10/18, 55.6%). The median age at the latest examination of 18 affected subjects was 62.5 years (range, 25–94).

### Onset and visual acuity

The median age of onset of 13 affected subjects with available records was 45.0 years (range, 15–77). One subject had a childhood onset at 15 years of age (1/13, 7.6%; Patient 16). Late onset of 45 years of age or later was reported in seven subjects (7/13, 53.8%; Patients 3, 8, 9, 10, 12, 15, 17).

The median best-corrected decimal visual acuity (VA) converted to the logarithm of the minimum angle of resolution (LogMAR) in the right and left eye of 18 affected subjects with available records was 0.52 (range, −0.08–2.00) and 0.40 (−0.18–1.70), respectively. Eight out of 18 subjects had relatively favourable VA (8/18, 44.4%, Patients 1, 2, 3, 7, 11, 12, 15, 17; 0.22 or better LogMAR units in the better eye), eight had intermediate VA (8/18, 44.4%, Patients 5, 6, 9, 10, 13, 14, 16, 18; between 0.22 and 1.0 LogMAR units in the better eye). There were eight eyes from six patients with poor VA (8/36, 22.2%, Patient 1-right, 3-right, 4-both, 5-left, 8-both, 14-left; 1.0 or worse LogMAR units).

### Retinal imaging and morphological findings

**Figure 2:**
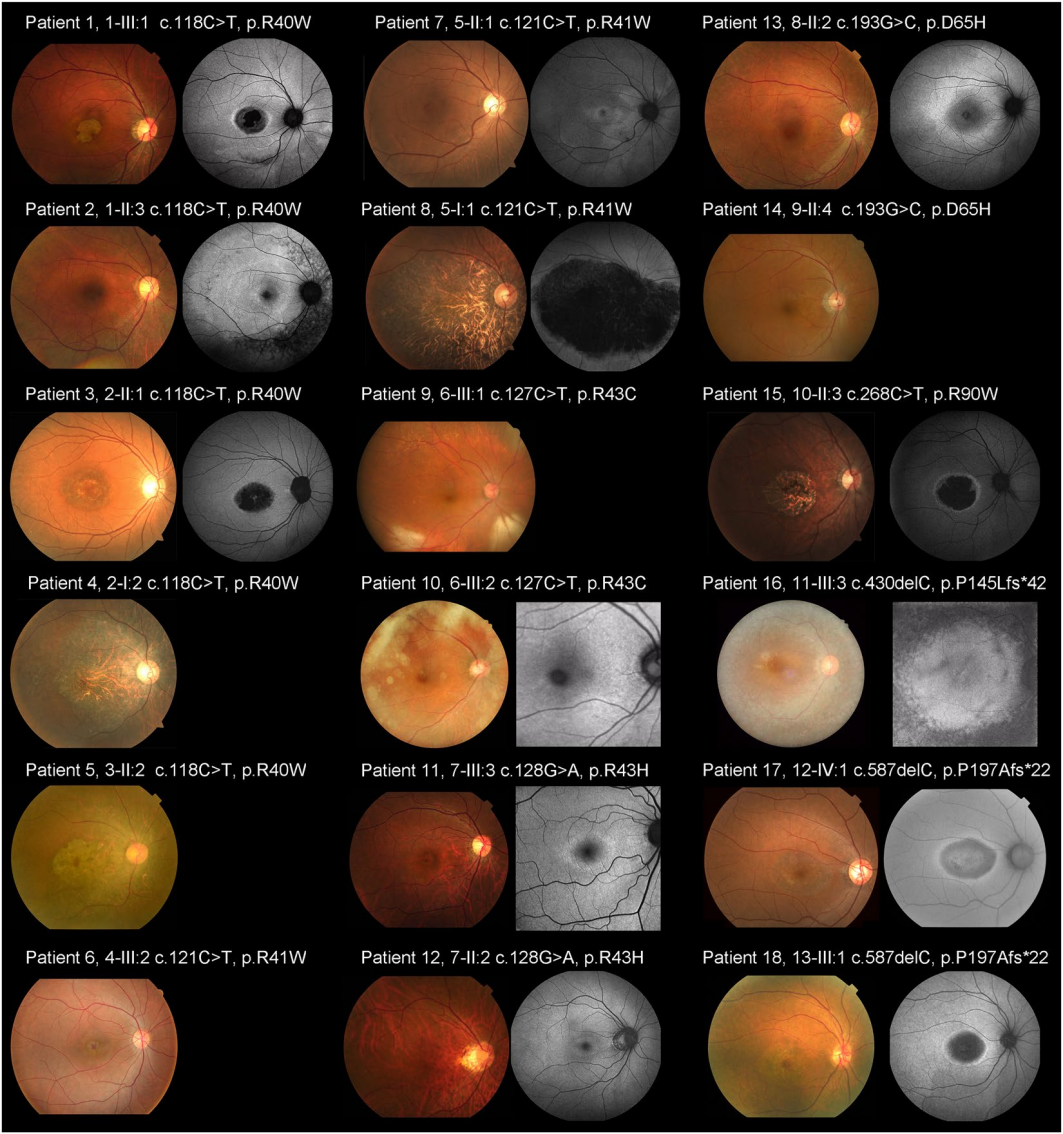
Fundus photographs and fundus autofluorescence images from 18 patients with *CRX*-associated retinal disorder (*CRX*-RD). Fundus photographs and fundus autofluorescence (FAF) images demonstrate macular atrophy in nine subjects (Patients 1, 3–6, 8, 15, 17, 18) and slight atrophic changes at the macula in three subjects (Patients 7, 11, 12). Peripheral atrophy is observed in four subjects (Patients 5, 13, 14, 16; detected by fundoscopy in Patients 5 and 14). Atrophic changes affecting the entire retina, including the macula, mid-periphery, and periphery are found in Patient 5. Macular atrophy is more evident on FAF images in eight subjects (Patients 1, 3, 8, 11, 12, 15, 17, 18). A ring of high density AF is observed in 11 subjects to various degrees (Patients 1, 2, 3, 7, 8, 11–13, 15, 17, 18). Foveal appearance is relatively preserved in nine subjects (Patients 1–3, 7, 10–12, 16, 17).

Fundus photographs were obtained in 18 affected subjects, and fundus autofluorescence (FAF) images were available in 13 affected subjects (Patients 1–3, 7, 8, 10–13, 15–18). Representative images are presented in Fig. 2. The detailed findings are described in Supplemental Table 1.

Marked macular atrophy was demonstrated in nine affected subjects (9/18, 50.0%; Patients 1, 3–6, 8, 15, 17, 18), and slight atrophic changes at the macula were found in three (3/18, 16.7%; Patients 7, 11, 12). Marked peripheral atrophy was observed in four subjects (4/18, 22.2%; Patients 5, 13, 14, 16). Marked atrophic changes along the arcade were noted in eight subjects (8/18, 44.4%; Patients 1, 2, 4, 5, 8, 10, 14, 16), and slight atrophic changes along the arcade were noted in two subjects (2/18, 11.1%; Patients 9, 13). Three of these ten subjects with atrophy along the arcade had isolated atrophy without macular or peripheral atrophy (Patients 2, 9, 10). One subject presented with atrophic changes affecting the entire retina, including the macula, mid-periphery, and periphery (1/18, 5.6%; Patient 5).

Retinal atrophy at the macula was more evident on FAF images in eight subjects (8/13, 61.5%; Patients 1, 3, 8, 11, 12, 15, 17, 18). A ring of high density AF was observed in 11 subjects to various degrees; eight with a ring that surrounded the macular changes (8/11, 72.7%, Patients 1, 3, 8, 11, 12, 15, 17, 18) and three with a ring that surrounded the mid-peripheral changes (3/11, 27.2%; Patients 2, 7, 13). Foveal appearance was relatively preserved in nine subjects (9/13, 69.2%; Patients 1–3, 7, 10–12, 16, 17).

**Figure 3:**
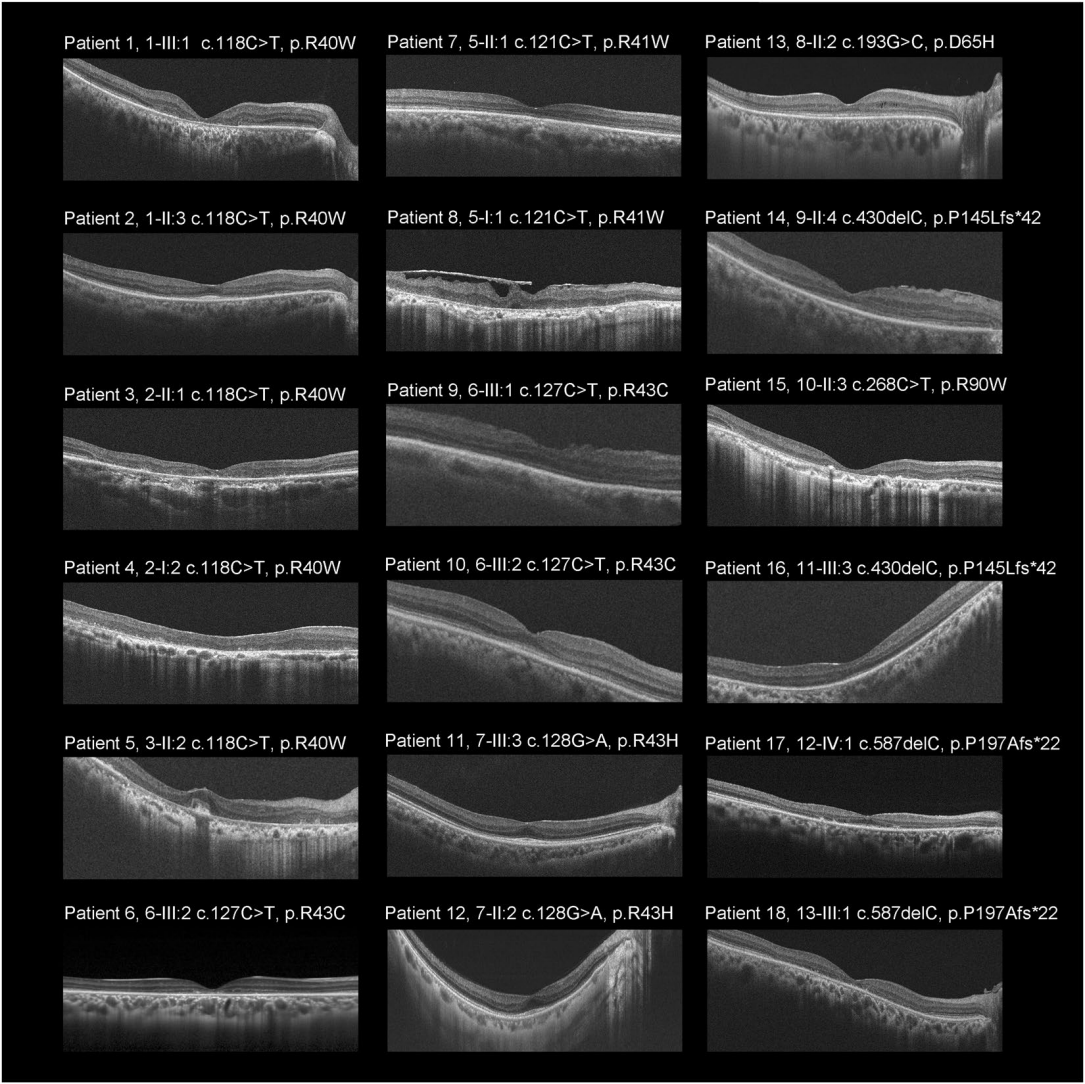
Spectral-domain optical coherence tomographic images from 18 patients with *CRX*-RD. Spectral-domain optical coherence tomographic images demonstrate outer retinal disruption at the macula in eight subjects (Patients 1, 3, 4–6, 8, 15, 18). Outer retinal disruption at the peri-macula is observed in 12 subjects (Patients 1–6, 8, 13–15, 17, 18). Intraretinal micro-cystic changes are noted in Patient 13. Epiretinal membrane is found in Patient 8. Marked preservation of the photoreceptor ellipsoid zone (EZ) line at the fovea is identified in eight subjects (Patients 2, 5, 7, 10–14), and slightly preserved EZ at the fovea isobserved in three subjects (Patients 9, 16, 17). Preserved foveal structure surrounded by parafoveal atrophy (i.e., bull’s eye pattern) is found in six subjects (Patients 1, 2, 10, 11, 12, 17).

Spectral-domain optical coherence tomography (SD-OCT) was obtained in 18 affected subjects, and representative images are presented in Fig. 3. Marked outer retinal disruption was demonstrated at the macula in eight subjects (8/18, 44.4%; Patients 1, 3, 4, 5, 6, 8, 15, 18). Outer retinal disruption in the peri-macula was observed in 12 subjects (12/18, 66.7%; Patients 1–6, 8, 13–15, 17, 18), and slight outer retinal disruption in the peri-macula was found in four subjects (4/18, 22.2%; Patients 10–12,16). Intraretinal micro-cystic changes and marked intraretinal fluid were noted in the right and left eyes, respectively, of Patient 13 (1/18, 5.6%).

Marked preservation of the photoreceptor ellipsoid zone (EZ) at the fovea was identified in eight subjects (8/18, 44.4%; Patients 2, 5, 7, 10–14), and slightly preserved EZ at the fovea was seen in three subjects (3/18, 16.7%; Patients 9, 16, 17). Preserved foveal structure surrounded by parafoveal atrophy (i.e., bull’s eye pattern) was observed in six subjects (6/18, 33.3%; Patient 1-left, 2-both, 10-both, 11-both, 12-both, 17-both).

### Visual fields and electrophysiological findings

The detailed findings of visual fields and electrophysiological findings are presented in Supplemental Table 2. Visual field testing was performed in 14 affected subjects (Patients 1, 3, 5, 7, 9–18). Central scotoma and paracentral scotoma were detected in eight subjects (8/14, 57.1%; Patients 1, 3, 5, 7, 9, 10, 15, 18). Paracentral scotoma without central scotoma was observed in five subjects (5/14, 35.7%; Patients 11, 13, 14, 16, 17). Peripheral visual field defects were found in 5 patients (5/14, 35.7%; Patients 9, 10, 13, 14, 16), two of whom also had central and paracentral scotoma (Patients 9, 10) and three had paracentral scotoma (Patients 13, 14, 16).

Full-field electroretinograms (ffERGs) were recorded in 16 affected subjects (Patients 1–3, 5–14, 16–18). Mildly decreased generalized light-adapted (LA) responses were demonstrated in six subjects (6/16, 37.5%; Patients 1, 3, 6, 7, 17, 18), five of whom had mildly decreased generalized dark-adapted (DA) responses (Patients 1, 3, 6, 7, 17). Severely decreased generalized LA and DA responses were detected in four subjects (4/16, 25.0%; Patients 8, 13, 14, 16), and moderately decreased generalized DA responses were found in one subject with unavailable LA responses (1/16, 6.3%; Patient 2). In two subjects, both generalized DA and LA responses were within normal limits (2/16, 12.5%; Patients 11, 12). A lower b to a ratio in DA bright flash responses (less than 0.9) was identified in seven subjects (7/16, 43.8%; Patients 2, 5, 6, 10–12, 17).

Multifocal ERGs (mfERGs) were recorded in four subjects (Patients 1, 9, 13, 18). Reductions in central responses were observed in three subjects (Patients 9, 13, 18), and a gross reduction in stimulus fields was found in one subject (Patient 1).

### Phenotype subgroups

Phenotype subgroup classification was performed in all 18 affected subjects. There were 13 subjects with CORD (13/18, 72.2%; Patients 1–10, 15, 17, 18), three with RP (3/18, 16.7%; Patients 13, 14, 16), and two with MD (2/18, 11.1%; Patients 11, 12). Intrafamilial differences in phenotypic subgroups among the affected subjects were not observed in all five families with multiple affected subjects (Families 1, 2, 5, 6, 7).

The mean age of onset of the two subjects with MD, 13 subjects with CORD, and three subjects with RP was 46.5 (range, 31–62), 50.3 (range, 30–77), and 26.0 (range, 15–37), respectively. The mean VA in the right/left eye of those with MD, CORD, and RP was 0.07/0.16 (range, −0.08–0.22/−0.08–0.4), 0.52/0.46 (range, 0.0–2.00/−0.18–1.7), and 0.68/0.55 (range, 0.52–0.82/0.4-light perception) LogMAR units, respectively.

### *CRX* variants

Variant data of 18 affected and 6 unaffected individuals from 13 families with *CRX*-RD are summarized in Supplemental Table 3. Seven heterozygous variants and one homozygous variant were identified by whole exome sequencing with target analysis of retinal disease-associated genes: c.118C > T, p.R40W; c.121C > T, p.R41W; c.127C > T, p.R43C; c.128G > A, p.R43H; c.268C > T, p.R90W; c.430delC, p.P145Lfs*42; c.587delC, p.P197Afs*22, and c.193G > C, p.D65H (NM_000554.5), respectively.

Five missense variants have been previously reported^16,27–32,34,36^. Three variants were reported in the heterozygous state: p.R40W for CORD, p.R41W for CORD, and p.R43C for CORD. One variant was previously reported in homozygous and heterozygous states: p.R90W homozygous for LCA and heterozygous for CORD^34,36^. One variant was previously reported only in the homozygous state: p.D65H for RP^29^. Three variants have never been reported: p.R43H, p.P145Lfs*42, and p.P197Afs*22. Co-segregation analysis was performed for four variants with the samples of unaffected family members; p.R41W (heterozygous), p.R43H (heterozygous), p.D65H (homozygous), and p.P145Lfs*42 (heterozygous, de novo). Four variants were recurrent in our cohort: p.R40W (heterozygous), p.R41W (heterozygous), p.D65H (homozygous), and p.P197Afs*22 (heterozygous).

Together with the clinical features of the affected subjects and the model of inheritance in the pedigree, eight disease-causing variants in the *CRX* gene were determined.

### *In silico* molecular genetic analysis

**Figure 4:**
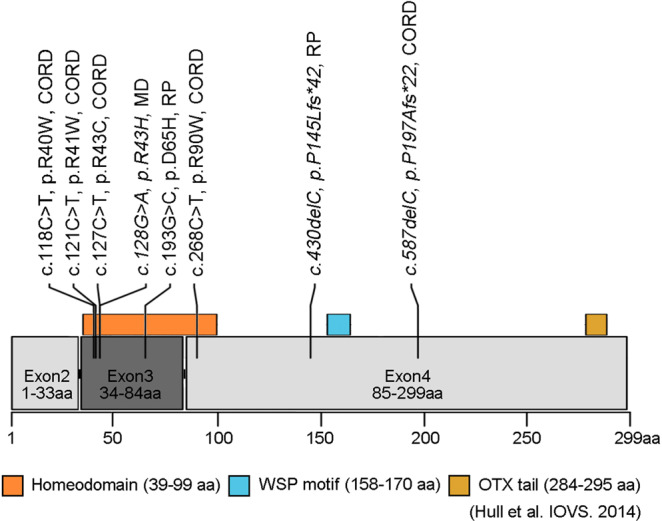
Schematic genetic and protein structures of CRX and the location of the detected variants. The *CRX* gene (ENST00000221996.7) contains four exons that encode a 299 amino acid protein containing a homeodomain, WSP motif, and OTX tail (Hull *et al*. 2014). Eight variants detected in this study are presented. Three novel variants are shown in Italics: p.R43H, p.P145Lfs*42, and p.P197Afs*22.

The detailed results of *in silico* molecular genetic analyses for the eight detected *CRX* variants are presented in Supplemental Tables 4 and 5. Schematic genetic and protein structures of *CRX* are shown in Fig. 4, and multiple alignment of seven species of *CRX* is presented in Supplemental Fig. 1.

Five missense variants were located within exon 3 (p.R40W, p.R41W, p.R43C, p.R43H, p.D65H) and the other missense variant (p.R90W) was in exon 4. Both exons are associated with the homeodomain of the CRX protein (residues 39–99; Fig. 4). Perfect evolutionary conservation was confirmed in five missense variants (p.R41W, p.R43C, p.R43H, p.D65H, p.R90W; Supplemental Fig. 1). Two truncating variants (p.P145Lfs*42 and p.P197Afs*22) were located in the last exon (exon 4), and nonsense-mediated decay did not appear to occur.

Allele frequency available for the five *CRX* variants (p.R41W, p.R43C, p.R43H, p.D65H, and p.R90W) in the general population of East Asia/South Asia/Africa/Europe (non-Finnish) was 0.0058%/0.0%/0.0%/0.00090%, 0.0%/0.0%/0.0%/0.0%, 0.0%/0.0%/0.0%/0.00090%, 0.0058%/0.0%/0.0%/0.0%, 0.0%/0.0%/0.0%/0.013%, respectively. Two variants had considerably higher allele frequencies in the East Asian population compared to the other populations (p.R41W and p.D65H).

General prediction, functional prediction, and conservation were assessed for the six missense variants and two single nucleotide deletion variants leading to frame shift and pathogenicity classification according to the American College of Medical Genetics and Genomics (ACMG) guidelines was performed. One pathogenic missense (p.R90W) and five likely pathogenic missense variants (p.R40W, p.R41W, p.R43C, p.R43H, and p.D65H) and the two likely pathogenic truncating variants (p.P145Lfs*42 and p.P197Afs*22) were revealed.

Overall, eight disease-causing variants in the *CRX* gene were identified in 13 families with ADCORD, ADRP, ADMD, and ARRP.

### Genotype-phenotype association

Genotype subgroup classification was performed in the proband of 13 families (Patients 1, 3, 5, 6, 7, 10, 11, 13–18). There were eight subjects in genotype subgroup A (heterozygous missense), two in genotype subgroup B (homozygous missense), and three in genotype subgroup C (truncating variants). A distribution of the 13 families based on genotype subgroups and phenotype subgroups is shown in Table 2. There is a trend, but the number is not sufficient to statistically demonstrate a significant genotype-phenotype association.

**Table 2:** Associations between genotype subgroups and phenotype subgroups in 13 families with CRX-RD.

	Genotype subgroup A (heterozygous missense)	Genotype subgroup B (homozygous missense)	Genotype subgroup C (heterozygous truncating)	Total
Phenotype subgroup A (MD)	1	0	0	1
Phenotype subgroup B (CORD)	7	0	2	9
Phenotype subgroup C (RP)	0	2	1	3
Total	8	2	3	13

Genotypic subgroup classification was performed based on the heterozygous/homozygous status of missense variants and presence of null variants (stop, frame shift, and splice site alteration): Genotype A–subjects with heterozygous missense variants; Genotype B–subjects with homozygous missense; and Genotype C–subjects with heterozygous truncating variants.

## Discussion

Detailed clinical and genetic characteristics of a Japanese cohort of 18 affected subjects from 13 families with *CRX*-RD are illustrated. Diverse clinical presentations with different inheritance patterns were identified in *CRX*-RD, including nine families with molecularly confirmed ADCORD, one family with ADMD, two families with ARRP, and one family with ADRP.

To our knowledge, these are data from the largest cohort of *CRX*-RD and includes the highest number of ADCORD cases to date, despite there being a well-characterized study of *CRX*-RD in 11 families from the UK^32^. Six out of 30 families (20.0%) diagnosed with CORD/MD/STGD and having a clear AD family history in the Japan Eye Genetics Consortium (JEGC) IRD cohort were associated with *CRX*-RD. The proportion of AD*CRX*-RD in molecularly confirmed ADCORD/MD/STGD in the JEGC cohort was considerably high (9/23 families, 39.1%) in comparison with European cohorts (e.g., 15.6% in the UK cohort)^7^. On the other hand, there were no families with LCA in our cohort, while four out of 11 families (36.4%) with *CRX*-RD in the UK cohort manifested the severe LCA phenotype. There could be a bias in the enrolment of IRD patients, although ethnic variation can also occur in CRX-RD.

The median age of onset for *CRX*-RD was in the fifth decade in our cohort, although it varied from teenage years to the 8^th^ decade, which is considerably later than that of other CORD/MD/STGD patients (e.g., 19.0 years for *ABCA4*-associated retinal disorder)^37^. In addition, over half of patients with late-onset disease (>45 years) have preserved favourable VA, and two maintained VA even after 10 years of disease history (Patients 13 and 15). Fundus and FAF showed variable findings; however, the severity of macular atrophy was generally associated with the severity of VA decline, and a characteristic ring of increased AF signal was observed in most subjects (>70%). Two-thirds of the subjects demonstrated preserved foveal structure often surrounded by parafoveal atrophy (i.e., bull’s eye pattern) with favourable VA, suggesting that late onset and morphological maintenance are indicators for preserved vision. These facts suggest that the severity and progression of visual impairment are mild in *CRX*-RD compared to that of CORD/MD/STGD caused by variants in other genes in the JEGC IRD cohort.

Electrophysiological findings of *CRX*-RD were also mildly affected in our cohort. Ten of 16 subjects (62.5%) had no or mild dysfunction both in generalized rod and cone systems, all of whom were classified into MD or CORD. In contrast, two subjects with CORD showed moderate retinal dysfunction, one subject with CORD and three subjects with RP had severe retinal dysfunction. Interestingly, a lower b to a ratio in DA bright flash responses was identified in approximately half of the subjects. Although an interaction with the phototransduction cascade was suggested in a previous study of CRX- and OTX2-transfected iris-derived cells^38^, the molecular mechanism to support this phenomenon is unknown. This electronegative finding was also observed in the early stage of other CORD/MD/STGD and was not specific for *CRX*-RD^13,39–41^; however, this characteristic feature can be helpful to consider *CRX*-RD in patients with early maculopathy.

Phenotype subgroups were associated with disease severity in our cohort. The subjects with MD had later-onset disease, maintained VA, and normal generalized retinal function. In general, the subjects with CORD had late-onset disease but VA decline, and the subjects with RP presented early-onset disease, VA decline, and severe generalized retinal dysfunction. Thus, determining phenotype subgroups with comprehensive clinical assessments provides crucial information directly related to disease severity and progression.

Eight pathogenic/likely pathogenic *CRX* variants were identified in our cohort, including three novel variants. One novel missense variant (p.R43H) located within the homeodomain of the CRX protein was found in two affected subjects with MD in a single family. A novel de novo truncating variant (p.P145Lfs*42) was revealed in a patient with early-onset RP. A novel recurrent truncating variant (p.P197Afs*22) was detected in two families with CORD. Comprehensive high-throughput gene screening of both affected and unaffected members was effective in obtaining a genetic diagnosis of *CRX*-RD manifesting AD or AR inheritance, as well as identifying de novo variants.

Four recurrent *CRX* variants were identified in our cohort, and two of these with available allele frequency in the general population revealed considerably high frequency in East Asia (p.R41W and p.D65H). Several cases with ADCORD caused by the former variant (p.R41W) have been reported in East Asian^16,28,35^, and the phenotype was the same as that observed in our two families (Families 4 and 5). Jin *et al*. reported only one Japanese RP case homozygous for the latter variant (p.D65H), and the phenotype was the same as that observed in our two families (Families 8 and 9)^29^. Given these facts, these two *CRX* variants with higher frequency are major causes of *CRX*-RD in the East Asian population, leading to CORD and RP, respectively.

The patients with heterozygous missense variants located within the homeodomain frequently associated with CORD (7/8 families; 87.5%) are consistent with previous studies^34^. A postulated dominant-negative effect can be considered for these heterozygous missense variants within the homeodomain, as reported for p.K88N^42^. Two families with homozygous missense variants (p.D65H) showed a severe phenotype, and the molecular mechanism is uncertain, unlike the well-studied homozygous missense variants (p.R90W), in which the mutant homeodomain showed a significantly reduced ability to transactivate the rhodopsin promoter and lower synergistic activation with the transcription factor NRL^36^. Three families with heterozygous truncating variants showed CORD (2/3, 66%) or RP (1/3, 33%). Notably, nonsense-mediated decay could possibly modify the phenotype in such variants^43^.

There are limitations in this study. The selection bias related to the disease severity should be inherent, since it is unusual for genetically affected subjects with good vision to visit clinics/hospitals. In addition, this cross-sectional retrospective case series study does not include longitudinal information; thus, natural history studies in a larger cohort could provide more accurate information regarding the disease progression of *CRX*-RD. The molecular mechanisms of AD missense, AR missense, and AD truncating variants have not yet been clarified in *CRX*-RD, and further functional investigation for each variant is required to conclude disease causation. The samples of affected and unaffected subjects of families with *CRX*-RD are still small to conclude the molecularly confirmed inheritance and genotype-phenotype associations/correlations in such a diverse disorder; thus, larger cohort studies are required for further analyses.

In conclusion, this large nationwide cohort study delineates the clinical and genetic characteristics of *CRX*-RD in Japan. A high proportion of AD*CRX*-RD was determined in Japan, which manifests late-onset ADCORD. The frequently found missense variants located within the homeodomain of the CRX protein can explain the mild phenotype of *CRX*-RD. In contrast, a relatively severe RP phenotype was associated with homozygous *CRX* missense variants in a small number of patients. This information will help to monitor and counsel patients, as well as design future therapeutic trials.

## Methods

The protocol of this study adhered to the tenets of the Declaration of Helsinki, which was approved by the ethics committee of the participating institutions from Japan: National Institute of Sensory Organs, National Hospital Organization Tokyo Medical Center (Reference; R18-029). Informed consent was received from all participants for the tests after an explanation of the procedures, and permission was obtained to use their medical data for research.

### Participants

Participants with a clinical diagnosis of IRD and available genetic data were studied between 2008 and 2018 as a part of the Japan Eye Genetics Consortium Studies (JEGC studies; http://www.jegc.org/)^44^. A total of 1294 subjects from 730 families registered to the JEGC cohort were surveyed, including 30 families with ADCORD/MD/STGD (defined as families with clear autosomal dominant family history).

### Clinical investigations

Medical history was obtained for all affected subjects and unaffected family members (where available). The onset of disease was defined as the age when any visual symptom was first noted by patients or parents or when the subject was first diagnosed.

Comprehensive ophthalmological examinations were performed in all affected subjects and unaffected family members (where available), including measurements of decimal VA converted to LogMAR units, ophthalmoscopy, fundus photography, FAF imaging, SD-OCT, kinetic and static visual field testing, and electrophysiological assessments according to the international standards of the International Society for Clinical Electrophysiology of Vision (ISCEV)^45–48^.

### Phenotype subgroups

For the purpose of this study, phenotype subgroups were defined based on clinical manifestations such as onset of disease, natural course, lesioned part on retinal imaging, and pattern of retinal dysfunction, partially according to a previous report^34^: LCA (including early-onset RP), a severe retinal dystrophy with early onset (<10 years) and extinguished retinal function; RP (including rod-cone dystrophy), a progressive retinal dystrophy often initially presenting peripheral atrophy with generalized rod dysfunction greater than cone dysfunction; CORD, a progressive retinal dystrophy often initially presenting macular atrophy with generalized cone dysfunction greater than rod dysfunction; MD, a progressive retinal dystrophy presenting macular atrophy with confined macular dysfunction despite no abnormalities in generalized cone and rod function.

### Genetic screening of the *CRX* gene

Genomic DNA was extracted from affected subjects and unaffected family members (where available for co-segregation analysis). Whole exome sequencing with target sequence analysis of 301 retinal disease-associated genes (based on RetNET; https://sph.uth.edu/retnet/home.htm; accessed on 1 July 2017) was performed according to a previously published method and through the Phenopolis platform (www.phenopolis.org)^44,49^. The identified variants were filtered on their allele frequency (less than 1%) in the Human Genetic Variation Database (HGVD; http://www.genome.med.kyoto-u.ac.jp/SnpDB/about.htm; accessed on 1 July 2017), which provides allele frequency of the general Japanese population. Depth and coverage for the target areas were examined with the integrative Genomics Viewer (http://www.broadinstitute.org/igv/) to detect structural variants.

Disease-causing variants were determined from the called/detected variants in the 301 retinal disease-associated genes, in consideration of the clinical findings of the affected subjects, the model of inheritance in the pedigree, and the results of co-segregation analysis.

### *In silico* molecular genetic analysis

The allele frequency of all detected *CRX* variants in the HGVD, Integrative Japanese Genome Variation (iJGVD 2k; https://ijgvd.megabank.tohoku.ac.jp/; accessed on 1 August 2018), 1000 genome (http://www.internationalgenome.org/; accessed on 1 August 2018), and the genome Aggregation Database (gnomAD) (http://gnomad.broadinstitute.org/; accessed on 1 August 2018)was established.

All detected *CRX* variants were analysed with prediction programs: MutationTaster (http://www.mutationtaster.org/; accessed on 1 August 2018), FATHMM (http://fathmm.biocompute.org.uk/9; accessed on 1 August 2018), SIFT (https://www.sift.co.uk/; accessed on 1 August 2018), PROVEAN (http://provean.jcvi.org/index.php; accessed on 1 August 2018), and Polyphen 2 (http://genetics.bwh.harvard.edu/pph2/; accessed on 1 August 2018). Evolutionary conservation scores were calculated for all detected *CRX* variants via the UCSC database (https://genome.ucsc.edu/index.html; accessed on 1 August 2018). Pathogenicity classification of all detected variants was performed based on the guidelines of the ACMG^50^.

### Genotype subgroups

Genotypic subgroup classification was performed based on the heterozygous/homozygous state of missense variants and presence of truncating variants: Genotype A–subjects with heterozygous missense variants; Genotype B–subjects with homozygous missense variants; and Genotype C–subjects with heterozygous truncating variants.

## Supplementary information

Supplemental Figure 1.

Supplemental Table 1.

Supplemental Table 2.

Supplemental Table 3.

Supplemental Table 4.

Supplemental Table 5.
