# Supplementary material for: Clinical and Genetic Characteristics of 18 Patients from 13 Japanese Families with CRX-associated retinal disorder: Identification of Genotype-phenotype Association
Source: Sci Rep. 2020 Jun 12;10:9531. doi: 10.1038/s41598-020-65737-z (PMC7293272; doi:10.1038/s41598-020-65737-z)
Supplement: Supplementary file 1 — Supplemental Figure 1. [file 41598_2020_65737_MOESM1_ESM.pdf]

(Title)

Clinical and Genetic Characteristics of 18 Patients from 13 Japanese Families with CRX-associated retinal disorder: Identification of Genotype-phenotype Association

(Author list)

Yu Fujinami-Yokokawa, Kaoru Fujinami, Kazuki Kuniyoshi, Takaaki Hayashi, Shinji Ueno, Atsushi Mizota, Kei Shinoda, Gavin Arno, Nikolas Pontikos, Lizhu Yang, Xiao Liu, Hiroyuki Sakuramoto, Satoshi Katagiri, Kei Mizobuchi, Taro Kominami, Hiroko Terasaki, Natsuko Nakamura, Shuhei Kameya, Kazutoshi Yoshitake, Yozo Miyake, Toshihide Kurihara, Kazuo Tsubota, Hiroaki Miyata, Takeshi Iwata, Kazushige Tsunoda; Japan Eye Genetics Consortium.

```
                                                                         R40 R41 R43
Homo_sapiens                   MMAYMNPGPHYSVNALALSGPSVDLMHQAVPYPSAPRKQRRERTTFTRSQLEELEALFAK
Mus_musculus                   MMAYMNPGPHYSVNALALSGPNVDLMHQAVPYSSAPRKQRRERTTFTRSQLEELEALFAK
Rattus_norvegicus              MMAYMNPGPHYSVNALALSGPSVDLMHQAVPYSSAPRKQRRERTTFTRSQLEELEALFAK
Xenopus_tropicalis             MMSY-IKQPHYAVNGLTLAGTGMDLLHSAVGYPTTPRKQRRERTTFTRAQLDILEALFAK
Bos_taurus                     MMAYMNPGPHYSVNALALSGPSVDLMHPAVSYPSAPRKQRRERTTFTRSQLEELEALFAK
Canis_lupus_familiaris         MMAYMNPGPHYSVNALALSGPSVDLMHQAVSYPSAPRKQRRERTTFTRSQLEELEALFAK
Callithrix_jacchus             MMAYMNPGPHYSVNTLALSGPSVDLMHQAVPYPSAPRKQRRERTTFTRSQLEELEALFAK
                               ** *    *** ** * * *   ** * ** *   ************* **  *******
                                   D65                      R90
Homo_sapiens                   TQYPDVYAREEVALKINLPESRVQVWFKNRRAKCRQQRQQQKQQQQPPGGQAKARPAKRK
Mus_musculus                   TQYPDVYAREEVALKINLPESRVQVWFKNRRAKCRQQRQQQKQQQQPPGAQTKARPAKRK
Rattus_norvegicus              TQYPDVYAREEVALKINLPESRVQVWFKNRRAKCRQQRQQQKQQQQPPGVQAKARPAKRK
Xenopus_tropicalis             TRYPDIFMREEVALKINLPESRVQVWFKNRRAKCRQQQQQSTGQAKPRPAKKKTSPARET
Bos_taurus                     TQYPDVYAREEVALKINLPESRVQVWFKNRRAKCRQQRQQQKQQQQPPGAQAKARPAKRK
Canis_lupus_familiaris         TQYPDVYAREEVALKINLPESRVQVWFKNRRAKCRQQRQQQKEQPQPPGAQTKARPAKRK
Callithrix_jacchus             TQYPDVYAREEVALKISLPESRVQVWFKNRRAKCRQQRQQQKQQQQPPGGQAKARPAKRK
                               * ***   ******** ******************** **   *  *     *  **

Homo_sapiens                   AGTSPRPSTDVCPDPLGISDSYSPPLPGPSGS-PTTAVATVSIWSPASESPLPEAQRAGL
Mus_musculus                   AGTSPRPSTDVCTDPLGISDSYSPSLPGPSGS-PTTAVATVSIWSPASEAPLPEAQRAGL
Rattus_norvegicus              AGTSPRPSTDVCTDPLGISDSYSPSLPGPSGS-PTTAVATVSIWSPASESPLPEAQRAGL
Xenopus_tropicalis             NS------------EASTNGQYSPPPPGTAVTPSSTASATVSIWSPASISPIPDPLSAAT
Bos_taurus                     AGTSPRSSTDVCPDPLGISDSYSPPLPGPSGS-PTTAVATVSIWSPASESPLPEAQRAGL
Canis_lupus_familiaris         AGMSPRSSSDVCPDPLGISDSYSPPLLGPSGS-PTTAVATVSIWSPASESPLPEAQRGGL
Callithrix_jacchus             AGTSPRPSTDVCPDPLGISDSYSPPLPGPSGS-PSTAVATVSIWSPASESPLPEAQRAGL
                                                    ***   *       ** **********  * *

Homo_sapiens                   VASGPSLTSAPYAMTYAPASAFCSSPSAYGSPSSYFSGLD--PYLSPMVPQLGGP--ALS
Mus_musculus                   VASGPSLTSAPYAMTYAPASAFCSSPSAYASPSSYFSGLD--PYLSPMVPQLGGP--ALS
Rattus_norvegicus              VASGSSLTSAPYAMTYAPASAFCSSPSAYASPSSYFSGLD--PYLSPMVPQLGGP--ALS
Xenopus_tropicalis             TPC--MQRSAGYPMTYSQAPAYT---QSYGGSSSYFTGLDCGSYLSPMHPQLSAPGATLS
Bos_taurus                     VASGPSLTSAPYAMTYAPASAFCSSPSAYGSPSSYFSGLD--PYLSPMVPPLGGP--ALS
Canis_lupus_familiaris         VASGPPLTSTPYAMTYAPASAFCSSPSAYGSPSSYFSGLD--PYLSPMVPQLGGP--ALS
Callithrix_jacchus             VASAPSLTSAPYAMTYAPASAFCSSPSAYGSPSSYFSGLD--PYLSPMVPQLGGP--ALS
                                       *  * ***  * *       *   **** ***   ***** * *  *   **

Homo_sapiens                   PLSGPSVGPSLAQSPTSLSGQSYGA----YSPVDSLEFKDPTGTWKFTYNPMDPLDYKDQ
Mus_musculus                   PLSGPSVGPSLAQSPTSLSGQSYST----YSPVDSLEFKDPTGTWKFTYNPMDPLDYKDQ
Rattus_norvegicus              PLSGPSVGPSLAQSPTSLSGQSYST----YSPVDSLEFKDPTGTWKFTYNPMDPLDYKDQ
Xenopus_tropicalis             PIATPTMGSHLSQSPASLSAQGYGASSLGFTSVDCLDYKDQTASWKLNFNTTDCLDYKDQ
Bos_taurus                     PLSGPSVGPSLTQSPTSLSGQSYGT----YSPVDSLEFKDPTGTWKFTYNPMDPLDYKDQ
Canis_lupus_familiaris         PLSGPSVGPSLAQSPTSLSGQSYGT----YSPVDSLEFKDPTGTWKFTYNPMDPLDYKDQ
Callithrix_jacchus             PLSGPSVGPSLAQSPTSLSGQSYGA----YSPVDSLEFKDPTGTWKFTYNPMDPLDYKDQ
                               *   *  *  * *** *** * *         ** *  ** *  **   *  * ******

Homo_sapiens                   SAWKFQIL
Mus_musculus                   SAWKFQIL
Rattus_norvegicus              SAWKFQIL
Xenopus_tropicalis             SSWKFQVL
Bos_taurus                     SAWKFQIL
Canis_lupus_familiaris         SAWKFQIL
                               ********
```

Supplemental Figure 1. Multiple alignment of eight species of CRX

The alignment was performed with the Clustal Omega program (https://www.ebi.ac.uk/Tools/msa/clustalo/; accessed on 1 August 2018), and the amino acid-sequence alignment is numbered in accordance with the Homo sapiens CRX sequence (ENST00000221996.7). An asterisk indicates perfect conservation across the seven species (only vertebrate models are available). The positions of variant residues are highlighted with a grey background: p.R40W, p.R41W, p.R43C, p.R43H, p.D65H, and p.R90W.
